# Bioinspired Bottlebrush Polymers for Aqueous Boundary Lubrication

**DOI:** 10.3390/polym14132724

**Published:** 2022-07-03

**Authors:** Xiaoyan Liu, Per M. Claesson

**Affiliations:** 1 School of Chemistry and Chemical Engineering, Shaanxi Normal University, Xi’an 710062, China; 2 Division of Surface and Corrosion Science, Department of Chemistry, School of Engineering Sciences in Chemistry, Biotechnology and Health, KTH Royal Institute of Technology, SE-100 44 Stockholm, Sweden; percl@kth.se

**Keywords:** bioinspired bottlebrush polymers, aqueous boundary lubrication, friction, wear resistance

## Abstract

An extremely efficient lubrication system is achieved in synovial joints by means of bio-lubricants and sophisticated nanostructured surfaces that work together. Molecular bottlebrush structures play crucial roles for this superior tribosystem. For example, lubricin is an important bio-lubricant, and aggrecan associated with hyaluronan is important for the mechanical response of cartilage. Inspired by nature, synthetic bottlebrush polymers have been developed and excellent aqueous boundary lubrication has been achieved. In this review, we summarize recent experimental investigations of the interfacial lubrication properties of surfaces coated with bottlebrush bio-lubricants and bioinspired bottlebrush polymers. We also discuss recent advances in understanding intermolecular synergy in aqueous lubrication including natural and synthetic polymers. Finally, opportunities and challenges in developing efficient aqueous boundary lubrication systems are outlined.

## 1. Introduction

The origin of friction is the energy dissipation processes that occur as two surfaces slide against each other [1]. Friction forces between two surfaces are often characterized by the effective friction coefficient, *μ*_eff_, that is, calculated by taking the ratio of friction force (*F*_Friction_) and applied load (*F*_Load_). The friction coefficient in synovial joints in mammals was found to be as low as 0.001 as measured by hip function simulator machines, even though values reported in different studies vary significantly [2]. It can be safely concluded that the friction coefficient is well below 0.01. For example, Gale et al. have reported values in the range of 0.002~0.006 [3]. This extremely efficient aqueous lubrication ability of the synovial joint is achieved by an association of lubricin, hyaluronan, phospholipids, and aggrecan [4,5,6]. Lubricin and aggrecan, bottlebrush-like biomacromolecules that have densely grafted pendant chains are abundant in synovial joints [7,8]. It is believed that these bottlebrush structured biomacromolecules play key roles for a number of critical biological functions, for example, hydration, aqueous boundary lubrication, wear resistance as well as mediating the rheological and mechanical properties under pressure [8,9].

Inspired by nature, synthetic bottlebrush polymers consisting of a linear polymeric backbone on which side chains are densely grafted have been designed and synthesized [10,11,12]. Theoretical studies and computer simulations have been used to predict the interfacial properties of bottlebrush polymer layers. The adsorbed amount of different bottlebrush polymers at the mica/silica surfaces has been predicted by lattice mean-field theory and compared with experimental data [13]. The frictional forces between polymer brush-like layers formed by linear polymers have been predicted by molecular dynamic simulations and scaling theory [14,15]. In addition, the interactions between bottlebrush polyelectrolyte layers have been predicted by molecular dynamic simulations [16]. In recent years, considerable progress has been made in theory and computer simulation to investigate the interfacial properties of polymer brush layers. For more detailed discussions, we recommend the valuable reviews on the theoretical studies of interaction forces and the lubrication properties of polymer brush-like layers [17,18].

The adsorption and interfacial lubrication properties between surfaces coated with a range of different bioinspired bottlebrush polymers have been extensively investigated by various surface analytical techniques [11,12,13,19,20,21,22,23,24,25,26,27,28,29,30,31,32,33,34,35,36,37,38,39,40,41,42,43,44,45,46,47,48,49,50,51]. Optical reflectometry [27,28,29], ellipsometry [52], neutron reflectometry [28,52], and quartz crystal microbalance with dissipation (QCM-D) [27,29] have been used to study the adsorption and desorption of bottlebrush polymers on surfaces in aqueous media. Direct force measurement techniques including surface force apparatus (SFA) [26,43,44,46], atomic force microscopy-colloidal probe technique (AFM-CP) [12,30], and mini traction machine [11,31] have been used to determine surface forces and/or friction forces between the bottlebrush polymer layers. Such measurements have shown that very efficient lubrication properties can be achieved by bottlebrush structured polymers [12,19,20,21,22,23,30,31,48,53]. Such excellent performance and highly promising lubrication systems are of great general interest, not least for relieving arthritis pain [54].

To introduce the subject of this review, we start with a short introduction of the molecular structures and functions of several important bio-lubricants found in the synovial fluid. Next, we discuss recent experimental investigations of the interfacial lubrication properties of bottlebrush polymer layers including friction forces and wear resistance. In addition, synergistic aqueous lubrication mediated by natural and bottlebrush polymers and small amphiphilic molecules is also discussed. Finally, outlooks for future research in and use of aqueous boundary lubrication systems are provided.

## 2. Bio-Lubricants

**Figure 1:**
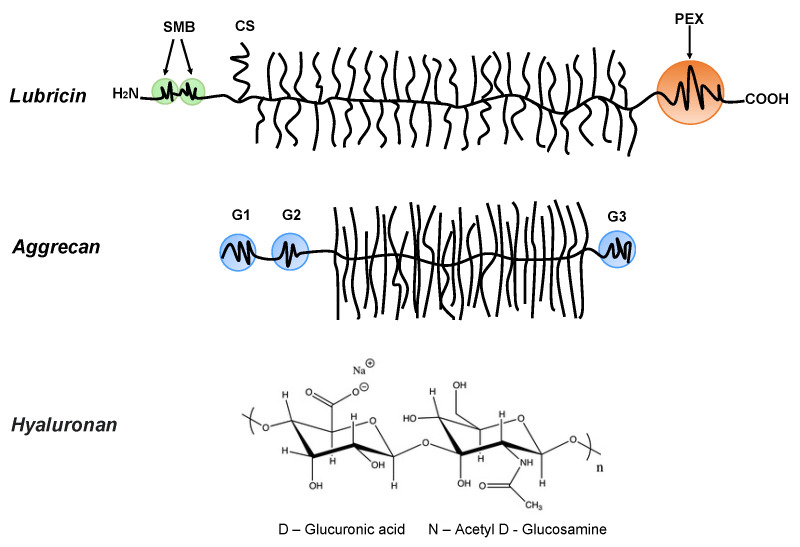
***Lubricin:*** containing the N-terminal 2-somatomedin B (SMB)-like domains and the C-terminal hemopexin (PEX)-like domain. Lubricin also contains a chondroitin sulfate (CS) side chain, and in the middle region, it has a densely glycosylated and mucin-like domain. ***Aggrecan***: containing three globular domains (G1, G2, and G3), and in the middle, it has a large extended domain heavily modified with glycosaminoglycans. ***Hyaluronan (HA)***: the anionic disaccharide building unit of hyaluronan.

Bio-lubricants including lubricin, hyaluronan, and phospholipids as well as aggrecan, which is important for the mechanical response of cartilage, play important roles in reducing friction forces and providing shock absorbing mechanical responses to articular cartilage surfaces [8]. These molecular structures are very different, but they share a common feature that they contain highly hydrophilic groups that bind water and allow for the hydration lubrication mechanism [55] to be operative. Lubricin is a glycoprotein with a bottlebrush structure. It is composed of the N-terminal by 2-somatomedin B (SMB)-like domains, the C-terminal with a hemopexin (PEX)-like domain, chondroitin sulfate (CS) side chain, and a densely glycosylated and mucin-like domain in the middle (Figure 1) [56,57]. It has been suggested that lubricin is able to counteract damages of the superficial zone of cartilage, and contributes to the preservation of chondrocytes in the joints [58]. The bottlebrush structure in lubricin and mucins also reduces the interpenetration zone, that is, the region where polymer chains on two opposing surfaces carrying such molecules overlap. This suggests that the bottlebrush structured polymers are promising candidates for reducing friction forces as the energy dissipation related to dragging polymer chains through the interpenetration zone is minimized. The interfacial lubrication studies of various types of mucins have shown that friction forces between surfaces can be reduced by mucins, which can be attributed to their extensive hydration, which arises from the large amount of oligomeric carbohydrate side chains [59,60,61,62,63,64]. Aggrecan also has a bottlebrush structure domain in the middle (Figure 1) [65,66]. The friction forces between surfaces with covalently attached cartilage aggrecan was found to be rather low (*μ_eff_* = 0.03 ± 0.01~0.11 ± 0.01, depending on the ionic strength of the solution) [67], even though this molecule’s most important function in the synovial joint area is found within the cartilage where it is associated with hyaluronan, and together with collagen, builds the intricate nanostructure of cartilage. For instance, the compressive resistance of articular cartilage has important contributions from hierarchical brush-on-brush structures formed by one hyaluronan associated with as many as 100 aggrecan molecules [68].

Hyaluronan and phospholipids also play important roles to achieve efficient aqueous lubrication systems [5,69,70,71,72,73,74,75]. Hyaluronan is a linear anionic polysaccharide (Figure 1). In fact, hyaluronan alone is not enough to reduce friction forces between surfaces [76,77]. However, hyaluronan is responsible for the viscous and elastic properties of the synovial fluid, which is very important for reducing the friction forces between articular cartilage [78,79]. It has also been reported that hyaluronan/aggrecan aggregates can achieve better lubrication than hyaluronan alone, which is due to the highly charged glycosaminoglycan segments on the aggrecan core protein [79]. In addition, the friction forces between damaged human cartilage can be reduced by self-assembled structures formed by hyaluronan and phospholipids [80]. The favorable interfacial lubrication properties of hyaluronan associated with phospholipids have also been observed in model systems [81,82,83]. The results of the friction force measurements between model silica surfaces coated with supported DPPC bilayers in the presence of hyaluronan have clearly indicated that the aggregates of hyaluronan/phospholipids are able to achieve low friction up to the pressure of 56 MPa, [81] which is higher than the pressure (25 ± 5.2 MPa) [84] that leads to the damage of the hyaline cartilage. Recently, multilayers have been prepared by the co-adsorption of HA and DPPC vesicles by Raj et al. [82], and the investigation showed that the friction coefficient between the layers was below 0.01 up to the pressure of 20 MPa. Over the last decade, Dedinaite and co-workers have investigated the intermolecular synergistic mechanism of the bio-lubricants in synovial fluid such as synergy pairs of hyaluronan and phospholipids [77,81,82,83,84,85,86,87]. Their studies indicate that synergistic lubrication can be achieved by the bio-lubricants working together. We summarize some of the observed synergistic effects of bio-lubricants regarding their interfacial lubricating properties in Table 1.

## 3. Bottlebrush Polymers

### 3.1. Synthesis of Bottlebrush Polymers

**Figure 2:**
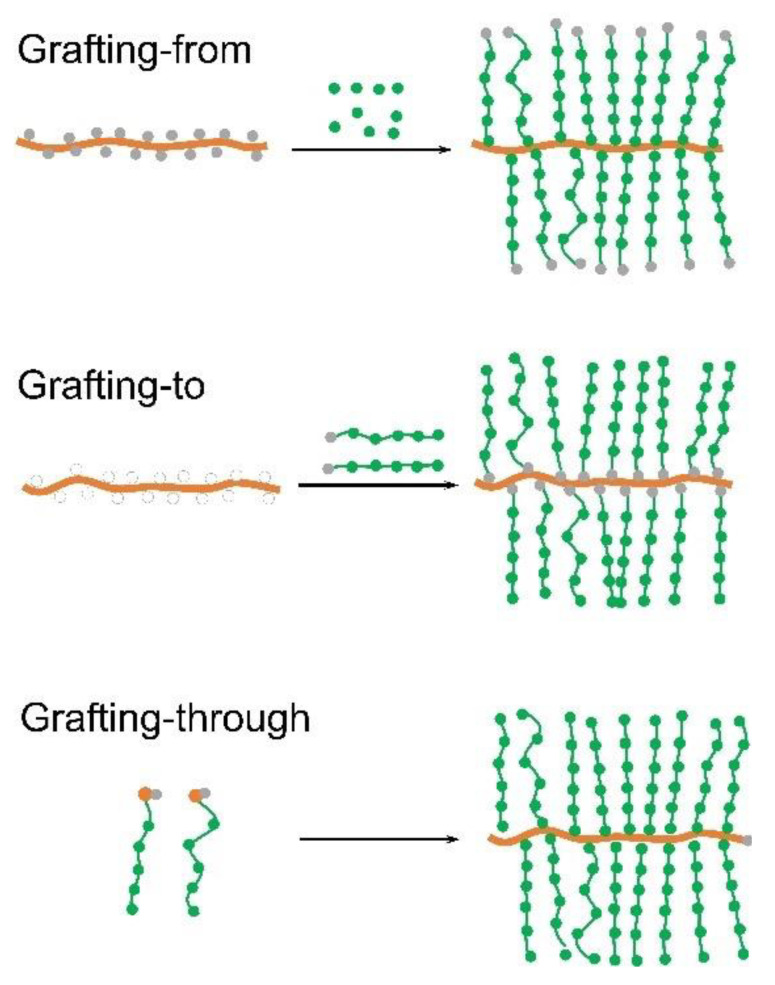
An illustration of three main synthetic approaches of bottlebrush polymer synthesis: grafting-from, grafting-to, and grafting-through.

Graft copolymers are composed of a polymeric backbone with densely grafted side chains, of which bottlebrush polymers are considered as a subset characterized by a very high grafting density. With increasing packing density of the side chains, a comb structure changes to a bottlebrush structure. This forces the backbone to adopt an extended conformation, and the molecule forms an overall cylindrical morphology. The conformation of the backbone and the length and the density of the side chains in bottlebrush polymers play significant roles in the lubrication properties. Inspired by bottlebrush bio-lubricants, synthetic bottlebrush polymers have been designed and prepared [91,92,93,94,95,96,97]. Conventional radical polymerization methods including atom transfer radical polymerization (ATRP), reversible addition-fragmentation chain transfer (RAFT), and nitroxide-mediated radical polymerization (NMP)) have been used to prepare precisely designed bottlebrush polymers [98,99,100,101,102,103]. According to how the side chains of the bottlebrush polymers are formed, the synthesis of bottlebrush polymers can be categorized into three approaches: (i) “grafting-from” (the polymerization of monomers from polyinitiators in the backbone) [99,100]; (ii) “grafting-through” (polymerization of macromonomers) [101,102]; and (iii) “grafting-to” (attachment of pre-formed side chains to the backbone) [104] (Figure 2). Novel bottlebrush polymers with advanced molecular structures such as double-brush [105], Janus [102], and core-shell [103] bottlebrush polymers have been prepared recently. Bottlebrush polymers can be applied in various fields such as templates of novel nanostructures, drug delivery, aqueous lubrication, and super-soft elastomers. In addition, novel cylindrical nanostructures such as segmented nanofibers [106] and anisotropic polymer nanostructures [107] have been developed. These emerging materials of precisely defined dimensions can be applied in many fields such as, sensors, bioimaging as well as lubrication [108,109]. Here, only a general overview of bottlebrush polymer synthesis is provided; for interested readers, we recommend a comprehensive review of the design and synthesis of bottlebrush polymers [10,92,93].

### 3.2. Adsorption of Bottlebrush Polyelectrolytes at Surfaces

**Figure 3:**
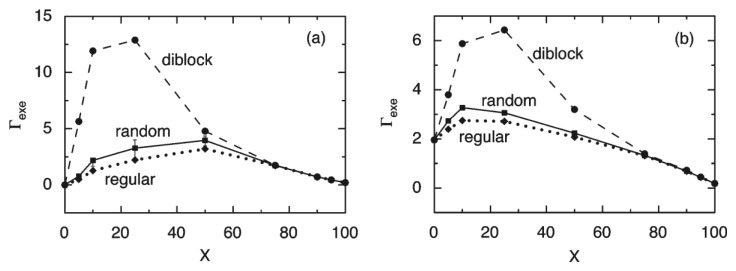
The theoretical surface excess (Γ_exe_) as a function of the percentage of charged segments, X, on the mica (**a**) and silica (**b**) surfaces with random (squares), regular (diamonds), and diblock (circles) distributions of the uncharged and charged main-chain segments. In panel a, the fluctuations of Γ_exe_ given as twice the standard deviation for 100 realizations of random distributions with the fixed fraction of charged segments are given as error bars. The figures were adopted with permission from [13]. Copyright © 2022 American Chemical Society.

The adsorption properties of bottlebrush polymers at the mica and silica surfaces have been predicted by the lattice mean-field theory by Linse and Claesson [13], Figure 3. The bottlebrush polymers were considered to consist of charged segments without side chains and uncharged segments with an attached side chain. The composition variable X stands for the percentage of charged segments ranging from X = 0 (uncharged bottlebrush polymer) to X = 100 (linear polyelectrolyte). The results of the theoretical modeling indicate that there is a large difference in the surface excess of bottlebrush polymers at the mica and silica surfaces. The difference in the surface excess between regular and random distributions is at most 20%, while a 5-fold difference in the surface excess between regular and diblock distributions has been predicted. These results indicate that the adsorption properties of bottlebrush polymers can be influenced by the polymer segment sequence, the non-electrostatic interactions as well as the surface charge density. To be able to tune the layer structure is important as the structure of the adsorbed polymer layers directly influences the interfacial lubrication properties of the polymer bearing surfaces.

Polymer bottlebrushes attached to surfaces can be prepared by either the “grafting-to” or the “grafting-from” method. The “grafting-to” method includes physical adsorption as well as the chemical covalent attachment of pre-prepared polymers. The “grafting-from” method is a bottom-up approach where polymer chains are grown by surface-initiated polymerization from a substrate [110]. Surfaces with anchored polymer bottlebrushes have shown great potential in lubrication applications [12,20,30,111,112,113,114,115,116]. Notably, the branched brush configuration and layers expressing polymer loop structures have been formed on surfaces by the “grafting-to” method through electrostatic interactions and using diblock bottlebrush polymers [30] and triblock bottlebrush polymers [116], respectively. The friction coefficients between surfaces coated with the bottlebrush polymer were as low as 10^−3^ up to the pressure of 2.1 MPa [116]. The excellent lubrication properties of these polymers arise from the very limited chain interpenetration of two opposing compressed bottlebrushes due to the strong steric repulsive interactions between the densely grafted side chains of the polymers. Furthermore, the side chains of the polymers are highly hydrated, allowing for strongly bound but easily sheared water layers (i.e., the hydration lubrication mechanism). In this context, it should be noted that shear properties of thin aqueous layers outside hydrophilic surfaces are very similar to that of bulk water down to thicknesses in the nanometer range as measured using a vertically oriented force sensor and AFM [117]. Polymer brushes on the surfaces can also be formed by linear polymers, and in many cases, favorable lubrication can be achieved. Interested readers are referred to the review of Kreer [18] and the references therein.

### 3.3. Interfacial Lubrication Properties of Bottlebrush Polymer Layers

The interfacial lubrication properties of the surfaces coated with a polymer layer depend on the chemical structure of the polymer, the structure of the polymer layer, and the water content of the polymer layer. These properties are influenced by several factors including the polymer–surface affinity as well as the solvent quality. The basis of aqueous boundary lubrication is the presence of a strongly bound yet easily sheared water layer, therefore, water-based lubricants always contain strongly hydrophilic regions. Thus, synthetic water-based lubricants can be designed following two criteria: (a) they shall be strongly hydrated to provide a low friction force; and (b) they shall adsorb strongly to the surface being lubricated to provide a high load bearing capacity. The latter criteria means that the polymers should remain on the surface under the action of a high load even during sliding motion. Considering this criterium, one is tempted to draw the conclusion that covalently attached polymers are to preferred over adsorbed ones. However, one should also consider that covalently attached layers do not self-heal when worn. In contrast, adsorbed layers can easily self-heal when the lubricating polymer is present in the surrounding bulk solution.

### 3.4. Random Bottlebrush Polymers

**Figure 4:**
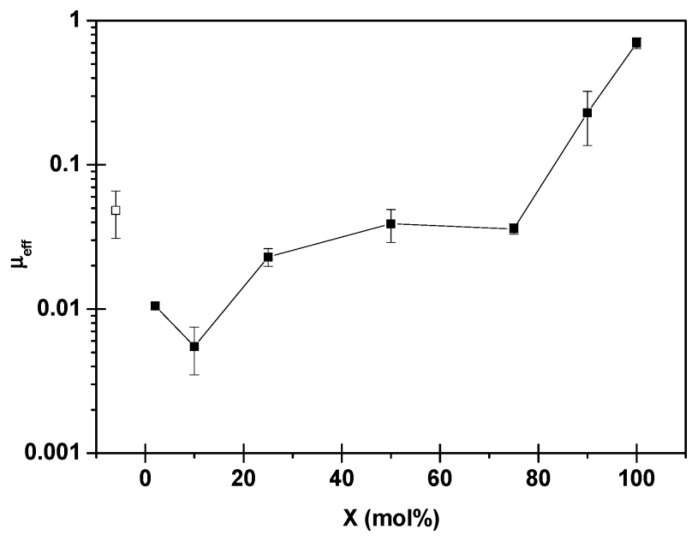
The effective friction coefficient (*μ*_eff_) of PEO_45_MEMA:METAC-*X* systems (filled squares), *μ*_eff_ of the bare mica–silica surface pair (unfilled square). The figures were adopted with permission from [12]. Copyright © 2022 American Chemical Society.

Bottlebrush polymers containing cationic segments with hydrophilic side chains have been developed, and investigations of interfacial lubrication have shown that such polymers can achieve a low friction force [11,12,29,31,36,48]. For instance, Pettersson et al. studied the effect of the side chain and charge density of random bottlebrush polyelectrolytes, poly(ethylene oxide)-methyl ether methacrylate:methacryloxyethyl trimethylammonium chloride-X (PEO_45_MEMA:METAC-*X*) by the AFM colloidal probe technique [12]. Here, X stands for the percentage of charged segments and 100-X is thus the percentage of segments carrying a 45 unit long poly(ethylene oxide) side chain. The effective friction coefficient (*μ*_eff_) of PEO_45_MEMA:METAC-*X* polyelectrolytes for the different systems is provided in Figure 4. Here, *μ*_eff_ was calculated by taking the ratio of friction force (*F*_Friction_) and applied load (*F*_Load_), at *F*_Load_ ≈ 7 nN, which was in the vicinity of the highest measured *F*_Load_. The results show that the PEO_45_MEMA:METAC-X polyelectrolytes with a high percentage of PEO_45_ segments, 100 ≤ 100 − X ≤ 25 (0 < X < 75), can achieve the lowest *μ*_eff_ at the mica–silica surface, implying that the frictional properties depend, to a significant degree, on which of the surfaces has the highest concentration of extended PEO_45_ segments.

The effects of the architectural parameters of poly(L-lysine)-*g*-poly(ethylene glycol) (PLL-*g*-PEG) including side chain (PEG) length, Lys/PEG grafting ratio, and backbone chain (PLL) length on the lubrication properties were studied by Spencer and co-workers [31,48]. These studies have shown how the interfacial lubrication properties of PLL-*g*-PEG can be optimized by varying the length of the side chains and the grafting ratio. The efficient aqueous boundary lubrication ability of PLL-*g*-PEG as well as PEO_45_MEMA:METAC-*X* was due to the densely grafted PEG side chains counteracting the interpenetration between the opposing layers under compression. The investigation of solvent quality on the lubricating ability of the surfaces coated with PLL-*g*-PEG has shown that the friction coefficient of the surfaces greatly increased with the decreasing solvent quality as the polymer layer structure changed from an expanded brush structure to a more random-coil-like structure [34,35]. In addition, the Scheutjens–Fleer self-consistent field theory has been employed to reveal how the architecture parameters, graft ratio, and graft length of random bottlebrush polymers influence the interaction between such polymers with a polyelectrolyte backbone and neutral hydrophilic side chains as well as an oppositely charged surface [118]. The modeling results indicated that the adsorption of random bottlebrush polymers is determined by the electrostatic and steric forces in the system, which can be affected by the graft ratio and graft length of the bottlebrush polymer. Particularly, it was demonstrated that the entropic penalty of adsorption increases as the side chains become longer.

### 3.5. Diblock Bottlebrush Polymers

**Figure 5:**
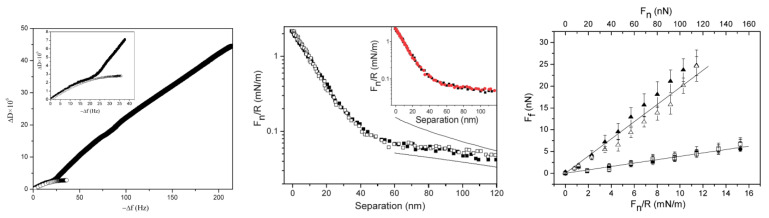
(**Left panel**) Dissipation change (Δ*D*) as a function of frequency change (*−*Δ*f*) upon adsorption of (METAC)_m_-*b*-(PEO_45_MEMA)_n_ (upper curve) and (PEO_45_MEMA)_n_ (lower curve) on silica. The inset shows the data in the range of *−*Δ*f* up to 40 Hz in more detail. The figures were adopted with permission from [29]. Copyright © 2022 American Chemical Society. (**Middle panel**) *F_n_/R* as a function of separation between the silica surfaces coated with (METAC)_m_-*b*-(PEO_45_MEMA)_n_. Fitted DLVO forces were obtained by using constant charge (upper line) and constant potential (lower line) boundary conditions. The inset shows the forces between the polymer layers prior to (black squares) and after (red circles) rinsing with water. (**Right panel**) Friction force (*F_f_*) as a function of load (*F_n_/R* and *F_n_*) of the bare silica surfaces in water (triangles) and after the adsorption of (METAC)_m_-*b*-(PEO_45_MEMA)_n_ in a 50 ppm polymer solution (the first cycle (squares) and the subsequent one (circles)). The straight lines were fitted to the data points obtained at low loads. The error bars corresponded to multiple friction force measurements. Filled and unfilled symbols represent the data points obtained on loading and unloading, respectively. The figures were adopted with permission from [30]. Copyright © 2022 The Royal Society of the Chemistry. In all cases, the polymer concentration of the aqueous solution was 50 ppm and the surface was silica.

Diblock bottlebrush polymers typically consist of one strongly adsorbing block and a second block with a bottlebrush structure with no or low tendency for adsorption. This polymer design is expected to provide an adsorbed layer structure with bottlebrush tails. Liu et al. studied the adsorption and frictional properties of diblock bottlebrush copolymers, (METAC)_m_-*b*-(PEO_45_MEMA)_n_, at the silica surfaces [29]. This diblock copolymer was composed of a cationic block and an uncharged bottlebrush block. The adsorbed amount of the diblock polymer on silica was high, and the thickness of the adsorbed polymer layer was found to be around 46 nm with a water content of around 90%. This result is in line with the prediction of the theoretical modeling in which diblock bottlebrush copolymers have a higher adsorbed amount than random and regular bottlebrush polymers at the silica surface [13] (Figure 3b). A change in the adsorbed layer structure was observed after a few hundred seconds of adsorption. This change can be illustrated by the QCM-D data by plotting the change in dissipation (Δ*D*) as a function of the change in frequency (Δ*f*) (left panel in Figure 5). The Δ*D* − Δ*f* curve for (METAC)_m_-*b*-(PEO_45_MEMA)_n_ initially followed that observed for the uncharged block ((PEO_45_MEMA)_n_), implying that initially, the diblock copolymer adsorbs parallel to the surface. However, for (METAC)m-*b*-(PEO_45_MEMA)_n_, the dissipation increased rapidly as the magnitude of the frequency change exceeded about 25 Hz. This means that (METAC)_m_-*b*-(PEO_45_MEMA)_n_ changes its orientation from preferentially parallel to the silica surface at low coverage (low dissipation) to significantly more extended conformations at high coverage (high dissipation).

The surface and friction forces of the silica surface coated with the (METAC)_m_-*b*-(PEO_45_MEMA)_n_ polymer were studied by the AFM colloidal probe technique, and some results are shown in the middle and right panels in Figure 5. The force curves between the silica surfaces coated with the diblock bottlebrush polymer were consistent with a double-layer force at separation beyond 40 nm. However, the force increased more steeply at separations below 40 nm, indicating the presence of a long-range steric repulsion. The calculated DLVO force curves indicated that the apparent double-layer potential of the adsorbed layer was found to be 33 mV at the onset of the steric interaction at a separation of 40 nm. In addition, no hysteresis was observed between the forces measured on approach and retraction. The friction coefficient was found to be 0.03 up to a pressure of 50 MPa, and thus the layer remained intact up to this high pressure [30]. The low friction force can be attributed to the strong steric repulsion and very limited interpenetration between the branched brush layers, combined with the strong hydration of the PEO side chains. Moreover, the friction forces between the adsorbed bottlebrush diblock polymer layers increased slightly more than predicted by Amontons’ rule at high applied loads, particularly in solutions with a high ionic strength. This suggests that another energy dissipative mechanism comes into play (e.g., some shear-induced lateral motion of the adsorbed molecules along the surface). However, the low friction forces were recovered when the applied load was reduced, indicating that attachments by electrostatic interaction may provide self-healing properties by re-adsorption or repositioning of the molecules desorbed or moved along the surface at high loads.

### 3.6. Triblock Bottlebrush Polymers

**Figure 6:**
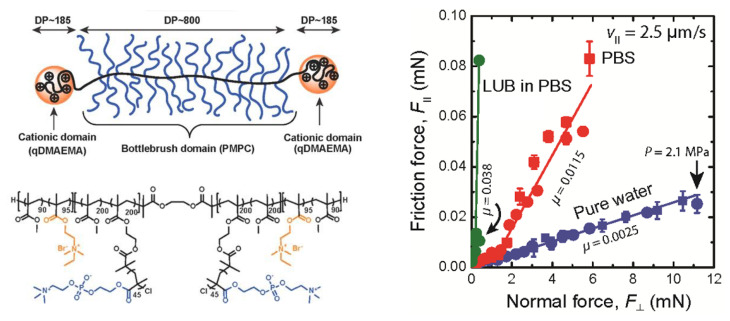
(**Left panel**) Schematic representation of the ABA triblock bottlebrush copolymer and the molecular structure of the triblock copolymer. (**Right panel**) Experimental results of the friction force vs. the normal force for the triblock bottlebrush adsorbed layer. The figures were adopted with permission from the study by Banquy et al. [116]. Copyright © 2022 American Chemical Society. The friction coefficient values of lubricin (LUB) in PBS in the right panel were adopted with permission from [119]. Copyright © 2022 Elsevier Inc.

Triblock bottlebrush polymers typically consist of two adsorbing end blocks and a middle block with a bottlebrush structure with no or low tendency for adsorption. Such a polymer design is interesting as it will facilitate formations of bottlebrush loops. Recently, an ABA triblock bottlebrush polymer, poly[(quaternized 2-(dimethylaminoethyl) methacrylate)-*co*-methyl methacrylate] (P(DMAEMA-*co*-MMA)), has been prepared by combining ATRP and post-modification techniques [116]. The triblock bottlebrush polymer was built by two positively charged domains with P(DMAEMA-*co*-MMA) chains (A blocks) and a bottlebrush region (B block), decorated with poly(2-methacryloyloxyethyl phosphorylcholine) (PMPC). The surface and friction forces between the surfaces coated with the triblock bottlebrush polymers were studied and the results showed that *μ*_eff_ of the adsorbed polymer layer was as low as 0.0025 up to a pressure of 2.1 MPa in pure water (Figure 6). The friction coefficient increased to 0.0115 ± 0.0003 in phosphate buffered saline (PBS) solution at 2.1 MPa due to the conformational changes in the outer part of the adsorbed polymer in the presence of salt, which was attributed to the increased affinity of the bottlebrush block to the surface and the screening of the electrostatic interaction between blocks adsorbed on the surface. A single loop adsorbed structure was formed by the triblock bottlebrush polymer in pure water, while in the presence of PBS, the polymer adopted to conformations with multiple loops and trains. The experimental data suggest that polymer loops are efficient in reducing friction forces as it reduces the interpenetration zone. This extremely low friction is additionally due to strong osmotic repulsion between the densely grafted side chains of the triblock bottlebrush polymers and the sliding of the surfaces is favored by the flow of water molecules during shearing.

### 3.7. Wear Resistance Influenced by Molecular Adsorption Strength

**Figure 7:**
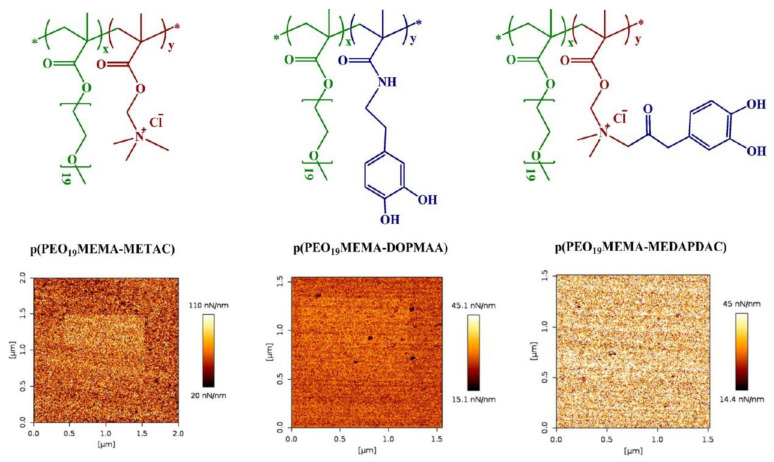
(**Top row**) The molecular structure of the catechol-based polymers. (**Bottom row**) Surface stiffness maps show the worn area in the middle of the AFM image. From left to right, at high loads (top of the worn area), wear was readily seen for electrostatic anchoring, less clearly seen when catechol groups were used, and not observable when both electrostatic anchoring and catechol groups were used. The figures were adopted with permission from [124]. Copyright © 2022 American Chemical Society.

The molecular adsorption strength cannot be judged by the polymer adsorbed amount. For example, polyelectrolytes typically attach more strongly to oppositely charged surfaces the higher their charge density. In contrast, the adsorbed amount typically decreases with polyelectrolyte charge density. When surfaces are modified with thin adsorbed polymer layers to form a well-lubricated surface, it is important that the adsorption strength is high to promote a long lifetime and function of the layer. The adsorption of the polymers onto surfaces may take place by non-specific electrostatic and hydrophobic interactions, hydrogen bonding, or specific anchoring groups. Particular attention has recently been given to mussel-inspired anchoring of polymers where catechol groups serve as anchors on the surfaces [120,121]. Catechol groups adsorb at most types of surfaces under wet condition, even on wet organic surfaces [122], and this provides a new approach to form efficient aqueous boundary lubrication layers with strong anchors. Self-healing properties of the adsorbed polymer layers have been observed in electrostatically anchored polymers systems [29,30,123]. Claesson et al. studied how different functional attachment mechanisms influence the adsorption strength by investigating molecular wear using statistical and diblock copolymers using different anchoring mechanisms, electrostatic (NH_3_^+^), hydrogen bonding/dispersion interaction (catechol), or both NH_3_^+^ and catechol [124,125]. It is difficult to observe wear scars of a sub-nanometer depth by using AFM. An alternative for soft adsorbed layers on hard surfaces is to determine the change in surface stiffness with increasing wear. An increase in stiffness implies that the thickness of the adsorbed polymer layer is reduced due to wear, and this effect originates from a larger contribution to the stiffness from the underlying substrate. It has been found that the adsorption strength varied as NH_3_^+^/catechol > catechol > NH_3_^+^ anchoring on silica surfaces in water [124] (Figure 7). This result implies that the anchoring of polymers containing catechol groups have a better ability to protect the layer from abrasion compared to electrostatic interactions alone. We note that anchoring the lubricant to the surface with the aid of electrostatic interactions has the disadvantage that the load-bearing capacity decreases with the increasing ionic strength of the solution. This situation can be improved by using non-electrostatically anchoring groups such as catechol.

### 3.8. Synergistic Aqueous Lubrication Mediated by Aggregates of Natural Molecules and Polymers

The interfacial frictional behavior of bottlebrush polymers associated with surfactants/phospholipids has been reported [50,126]. The interactions between a co-adsorbed poly(acrylic acid)-poly(acrylamide) diblock copolymer with cationic surfactant layers on mica surfaces were investigated by Drummond et al., who found that the lubrication ability of the surface could be improved by the associated structure, which was attributed to the hemifusion instability of the adsorbed surfactant layers being inhibited by the copolymer [126]. Furthermore, the aqueous boundary lubrication properties of complexes of the anionic surfactant (sodium dodecyl sulfate) with positively charged polymers (poly[3-(2-methyl propionamido)-propyl]trimethylammonium chloride (PMAPTAC)) have been investigated [50]. The study showed that the friction force was very low, even at pressures up to 20 MPa, except for some friction peaks that occurred due to the load and shear-induced structural changes in the layer. The data clearly demonstrated that the association of the polymer and surfactant had a much better aqueous boundary lubrication performance than the polymer or surfactant alone. This result implies that the internal organization of the aggregate is essential for developing efficient polyelectrolyte–surfactant systems for aqueous lubrication. This study provides a new approach to develop efficient water-based lubrication systems formed by synergistic self-assembly at solid/aqueous interfaces by utilizing the self-assembly structures of polyelectrolytes and surfactants.

Faivre et al. studied the lubrication and wear protection of a mixture of bottlebrush polymers and linear polymers (hyaluronan, HA) and found that this mixture provided wear protection both in water and saline [43]. This was attributed to the synergy of the two polymers by which a boundary film was formed by entanglements of the bottlebrush polymer and HA. Faivre et al. also studied the effects of polyzwitterionic bottlebrush polymer architecture such as monoblock, diblock, and triblock on the wear resistance, where both individual bottlebrush polymers and mixture of the bottlebrush polymers with HA were investigated [46]. The results demonstrated that the rupture pressure (P*) (i.e., the load bearing capacity) of the thin film formed by the bottlebrush polymers alone increased with the increasing content of the adhesive blocks on the bottlebrush polymers, P*_triblock_ > P*_diblock_ > P*_monoblock_. They suggested that this is because the monoblock polymer is rather peculiar since it has no terminal adhesive end blocks, the diblock polymer has similar conformation as the monoblock polymer, but also electrostatic interactions with the surface, while the triblock polymer tends to form loop structures that provide stronger anchoring to the surface and lower chain interpretation. In addition, the rupture pressure greatly increased when adding high-molecular weight HA into the bottlebrush polymer layers. This high protective performance was attributed to the strong intermolecular interaction between the bottlebrush polymers and HA. This study provides a new approach to enhance the interfacial lubrication and wear resistance of surfaces by using bottlebrush polymers and linear bio-lubricants, opening new routes toward the development of efficient aqueous boundary lubrication systems.

## 4. Outlook

An excellent aqueous boundary lubrication system will have a low energy dissipation when the opposing layers slide against each other. This means that the interpenetration zone should be small and the outer layer should be highly hydrated to allow for hydration lubrication. The wear resistance of the lubricating layer should also be considered as it is important to have a high load bearing capacity. This means that the lubricant should be strongly anchored to the surface, and here, catechol groups are a promising alternative to electrostatic anchoring. It is also important to have an efficient self-healing ability, which can be achieved by physical adsorption and by having a reservoir of the lubricants available in bulk solution. Several studies have demonstrated that very efficient aqueous boundary lubrication systems can be formed by tuning the structure of bottlebrush polymers, the layer conformation of bottlebrush polymers, and the molecular adsorption strength. Furthermore, highly efficient intermolecular synergistic lubrication has been achieved by aggregates of natural or synthetical molecules, for instance, HA/aggrecan, HA/phospholipids, COMP/lubricin, HA/lubricin/Type II collagen, cross-linked HA/DOPC, polyelectrolyte/surfactant, and HA/bottlebrush polymer. These results have shown that efficient lubrication can be achieved as different types of molecules work together. However, more observations are needed to reveal the internal structure of the aggregates, leading to synergistic lubrication in polymer–surfactant, polymer–polymer, and polymer–bio-lubricant systems. It is thus important to understand the intermolecular synergy mechanisms in lubrication and we hope that future studies of water-based lubrication will pay increasing attention to intermolecular synergies. Moreover, the wear resistance of the lubricating layer is greatly influenced by the molecular adsorption strength. Thus, future studies focusing on the wear resistance of both the lubricating layer and the underlying surfaces should be of high value. In addition, modeling studies are needed to optimize the polymer design to achieve excellent lubrication for surfaces of different chemical composition and surface roughness, which would provide an additional molecular understanding of the aqueous lubrication mechanism [55,117]. These related studies may not only provide new approaches to aqueous lubrication processes in technical systems but also lead to the development of new treatments and technologies to alleviate pain and prevent cartilage degeneration. The review discusses the significance and effects of the bottlebrush polymer design for excellent lubrication properties and wear resistance and summarizes the recent observations of the lubrication properties of bottlebrush polymers. We predict that future studies will provide answers to the fundamental questions and lead to the development of new technologies for aqueous lubrication systems, for instance, smart lubricating coatings with anti-fouling and anti-corrosion properties as well as tissue-engineering scaffolds.

## Figures and Tables

**Table 1:** The lubrication performance of the bio-lubricant aggregates. The table provides data for the effective friction coefficient, *µ*_eff_, and the highest pressure, *P*, investigated.

Compositions	Substrate	Interfacial Lubricating Properties	Ref.
HA + DPPC liposomes	Damaged human cartilage	The reduction in friction was 69.5%, *P* = 1.3 MPa	[79]
HA + Aggrecan	Mica	*μ_eff_* = 0.01, *P* = 1.6 MPa	[78]
HA + DPPC vesicles	Macroscopic glass surfaces	*μ_eff_* = 0.1, *P* = 210 MPa	[77]
HA + DPPC bilayer	Silica	*μ_eff_* = 0.03, *P* = 56 MPa	[80]
HA + DPPC vesicles	Silica	*μ_eff_* < 0.01, *P* = 23 MPa	[81]
COMP + lubricin	PMMA	*μ_eff_* = 0.06, *P* = 7 MPa	[85]
cross-linked HA + DOPC	Mica	*μ_eff_ >* 0.5, *P* = 2 MPa	[88]
HA + Lubricin	Mica	*μ_eff_* = 0.09–0.4, *P* = 4 MPa	[89]
HA + Lubricin + Type II collagen	Gold versus SiO_2_	*μ_eff_* = 0.01, *P* = 0.013 MPa	[90]

## Data Availability

The data presented in this study are available on request from the corresponding author.

## References

[1] Singer I.L., Pollock H. (2012). Fundamentals of Friction: Macroscopic and Microscopic Processes.

[2] Forster H., Fisher J. (1996). The Influence of Loading Time and Lubricant on the Friction of Articular Cartilage. Proc. Inst. Mech. Eng. Part H J. Eng. Med..

[3] Gale L.R., Coller R., Hargreaves D., Hills B.A., Crawford R. (2007). The role of SAPL as a boundary lubricant in prosthetic joints. Tribol. Int..

[4] Wright V., Dowson D. (1976). Lubrication and cartilage. J. Anat..

[5] Hills B.A. (2002). Surface-active phospholipid: A Pandora’s box of clinical applications. Part II. Barrier and lubricating properties. Intern. Med. J..

[6] Klein J. (2006). Molecular mechanisms of synovial joint lubrication. Proc. Inst. Mech. Eng. Part J J. Eng. Tribol..

[7] Levangie P.K., Norkin C.C. (2011). Joint Structure and Function: A Comprehensive Analysis.

[8] Dėdinaitė A. (2012). Biomimetic lubrication. Soft Matter.

[9] Abbasi M., Faust L., Wilhelm M. (2019). Comb and Bottlebrush Polymers with Superior Rheological and Mechanical Properties. Adv. Mater..

[10] Verduzco R., Li X.Y., Pesek S.L., Stein G.E. (2015). Structure, function, self-assembly, and applications of bottlebrush copolymers. Chem. Soc. Rev..

[11] Lee S., Muller M., Ratoi-Salagean M., Vörös J., Pasche S., De Paul S.M., Spikes H.A., Textor M., Spencer N.D. (2003). Boundary Lubrication of Oxide Surfaces by Poly(L-lysine)-g-poly(ethylene glycol) (PLL-g-PEG) in Aqueous Media. Tribol. Lett..

[12] Pettersson T., Naderi A., Makuška R., Claesson P.M. (2008). Lubrication Properties of Bottle-Brush Polyelectrolytes: An AFM Study on the Effect of Side Chain and Charge Density. Langmuir.

[13] Linse P., Claesson P.M. (2009). Modeling of Bottle-Brush Polymer Adsorption onto Mica and Silica Surfaces. Macromolecules.

[14] Galuschko A., Spirin L., Kreer T., Johner A., Pastorino C., Wittmer J., Baschnagel J. (2010). Frictional Forces between Strongly Compressed, Nonentangled Polymer Brushes: Molecular Dynamics Simulations and Scaling Theory. Langmuir.

[15] Spirin L., Galuschko A., Kreer T., Johner A., Baschnagel J., Binder K. (2010). Polymer-brush lubrication in the limit of strong compression. Eur. Phys. J. E.

[16] Russano D., Carrillo J.-M.Y., Dobrynin A.V. (2011). Interaction between Brush Layers of Bottle-Brush Polyelectrolytes: Molecular Dynamics Simulations. Langmuir.

[17] Leermakers F., Zhulina E., Borisov O. (2017). Interaction forces and lubrication of dendronized surfaces. Curr. Opin. Colloid Interface Sci..

[18] Kreer T. (2016). Polymer-brush lubrication: A review of recent theoretical advances. Soft Matter.

[19] Sakakibara K., Maeda K., Yoshikawa C., Tsujii Y. (2021). Water Lubricating and Biocompatible Films of Bacterial Cellulose Nanofibers Surface-Modified with Densely Grafted, Concentrated Polymer Brushes. ACS Appl. Nano Mater..

[20] Yan W., Ramakrishna S.N., Spencer N.D., Benetti E.M. (2019). Brushes, Graft Copolymers, or Bottlebrushes? The Effect of Polymer Architecture on the Nanotribological Properties of Grafted-from Assemblies. Langmuir.

[21] Sun Z., Feeney E., Guan Y., Cook S.G., Gourdon D., Bonassar L.J., Putnam D. (2019). Boundary mode lubrication of articular cartilage with a biomimetic diblock copolymer. Proc. Natl. Acad. Sci. USA.

[22] Adibnia V., Olszewski M., De Crescenzo G., Matyjaszewski K., Banquy X. (2020). Superlubricity of Zwitterionic Bottlebrush Polymers in the Presence of Multivalent Ions. J. Am. Chem. Soc..

[23] Jia W., Tian J., Bai P., Li S., Zeng H., Zhang W., Tian Y. (2018). A novel comb-typed poly(oligo(ethylene glycol) methylether acrylate) as an excellent aqueous lubricant. J. Colloid Interface Sci..

[24] Wei Q., Fu T., Yue Q., Liu H., Ma S., Cai M., Zhou F. (2021). Graphene oxide/brush-like polysaccharide copolymer nanohybrids as eco-friendly additives for water-based lubrication. Tribol. Int..

[25] Claesson P., Makuska R., Varga I., Meszaros R., Titmuss S., Linse P., Pedersen J.S., Stubenrauch C. (2010). Bottle-brush polymers: Adsorption at surfaces and interactions with surfactants. Adv. Colloid Interface Sci..

[26] Naderi A., Makuška R., Claesson P.M. (2008). Interactions between bottle-brush polyelectrolyte layers: Effects of ionic strength and oppositely charged surfactant. J. Colloid Interface Sci..

[27] Olanya G., Iruthayaraj J., Poptoshev E., Makuska R., Vareikis A., Claesson P.M. (2008). Adsorption Characteristics of Bottle-Brush Polymers on Silica: Effect of Side Chain and Charge Density. Langmuir.

[28] Liu X., Dedinaite A., Nylander T., Dabkowska A.P., Skoda M., Makuska R., Claesson P.M. (2014). Association of anionic surfactant and physisorbed branched brush layers probed by neutron and optical reflectometry. J. Colloid Interface Sci..

[29] Liu X., Dedinaite A., Rutland M., Thormann E., Visnevskij C., Makuska R., Claesson P.M. (2012). Electrostatically Anchored Branched Brush Layers. Langmuir.

[30] Liu X., Thormann E., Dedinaite A., Rutland M., Visnevskij C., Makuska R., Claesson P.M. (2013). Low friction and high load bearing capacity layers formed by cationic-block-non-ionic bottle-brush copolymers in aqueous media. Soft Matter.

[31] Müller M., Lee S., Spikes H.A., Spencer N.D. (2003). The Influence of Molecular Architecture on the Macroscopic Lubrication Properties of the Brush-Like Co-polyelectrolyte Poly(L-lysine)-*g*-poly(ethylene glycol) (PLL-*g*-PEG) Adsorbed on Oxide Surfaces. Tribol. Lett..

[32] Dedinaite A., Thormann E., Olanya G., Claesson P.M., Nyström B., Kjøniksen A.-L., Zhu K. (2010). Friction in aqueous media tuned by temperature-responsive polymer layers. Soft Matter.

[33] Yan X., Perry S.S., Spencer N., Pasche S., De Paul S.M., Textor M., Lim M.S. (2003). Reduction of Friction at Oxide Interfaces upon Polymer Adsorption from Aqueous Solutions. Langmuir.

[34] Müller M.T., Yan X., Lee S., Perry S.S., Spencer N.D. (2005). Preferential Solvation and Its Effect on the Lubrication Properties of a Surface-Bound, Brushlike Copolymer. Macromolecules.

[35] Müller M.T., Yan X., Lee S., Perry S.S., Spencer N.D. (2005). Lubrication Properties of a Brushlike Copolymer as a Function of the Amount of Solvent Absorbed within the Brush. Macromolecules.

[36] Lee S., Spencer N.D. (2007). Poly(l-lysine)-graft-poly(ethylene glycol): A versatile aqueous lubricant additive for tribosystems involving thermoplastics. Lubr. Sci..

[37] Hartung W., Drobek T., Lee S., Zürcher S., Spencer N.D. (2008). The Influence of Anchoring-Group Structure on the Lubricating Properties of Brush-Forming Graft Copolymers in an Aqueous Medium. Tribol. Lett..

[38] Lee S., Spencer N.D. (2008). Adsorption Properties of Poly(l-lysine)-*graft*-poly(ethylene glycol) (PLL-*g*-PEG) at a Hydrophobic Interface: Influence of Tribological Stress, pH, Salt Concentration, and Polymer Molecular Weight. Langmuir.

[39] Perrino C., Lee S., Choi S.W., Maruyama A., Spencer N.D. (2008). A Biomimetic Alternative to Poly(ethylene glycol) as an Antifouling Coating: Resistance to Nonspecific Protein Adsorption of Poly(l-lysine)-*graft*-dextran. Langmuir.

[40] Iruthayaraj J., Olanya G., Claesson P.M. (2008). Viscoelastic Properties of Adsorbed Bottle-brush Polymer Layers Studied by Quartz Crystal Microbalance—Dissipation Measurements. J. Phys. Chem. C.

[41] Perrino C., Lee S., Spencer N.D. (2008). End-grafted Sugar Chains as Aqueous Lubricant Additives: Synthesis and Macrotribological Tests of Poly(l-lysine)-graft-Dextran (PLL-g-dex) Copolymers. Tribol. Lett..

[42] Bijelic G., Shovsky A., Varga I., Makuska R., Claesson P.M. (2010). Adsorption characteristics of brush polyelectrolytes on silicon oxynitride revealed by dual polarization interferometry. J. Colloid Interface Sci..

[43] Faivre J., Shrestha B.R., Burdynska J., Xie G., Moldovan F., Delair T., Benayoun S., David L., Matyjaszewski K., Banquy X. (2017). Wear Protection without Surface Modification Using a Synergistic Mixture of Molecular Brushes and Linear Polymers. ACS Nano.

[44] Faivre J., Shrestha B.R., Xie G., Delair T., David L., Matyjaszewski K., Banquy X. (2017). Unraveling the Correlations between Conformation, Lubrication, and Chemical Stability of Bottlebrush Polymers at Interfaces. Biomacromolecules.

[45] Faivre J., Montembault A., Sudre G., Shrestha B.R., Xie G., Matyjaszewski K., Benayoun S., Banquy X., Delair T., David L. (2018). Lubrication and Wear Protection of Micro-Structured Hydrogels Using Bioinspired Fluids. Biomacromolecules.

[46] Faivre J., Shrestha B.R., Xie G., Olszewski M., Adibnia V., Moldovan F., Montembault A., Sudre G., Delair T., David L. (2018). Intermolecular Interactions between Bottlebrush Polymers Boost the Protection of Surfaces against Frictional Wear. Chem. Mater..

[47] Xia Y., Adibnia V., Huang R., Murschel F., Faivre J., Xie G., Olszewski M., De Crescenzo G., Qi W., He Z. (2019). Biomimetic Bottlebrush Polymer Coatings for Fabrication of Ultralow Fouling Surfaces. Angew. Chem. Int. Ed..

[48] Perry S.S., Yan X., Limpoco F.T., Lee S., Müller M., Spencer N.D. (2009). Tribological Properties of Poly(l-lysine)-*graft*-poly(ethylene glycol) Films: Influence of Polymer Architecture and Adsorbed Conformation. ACS Appl. Mater. Interfaces.

[49] Hartung W., Rossi A., Lee S., Spencer N.D. (2009). Aqueous Lubrication of SiC and Si3N4 Ceramics Aided by a Brush-like Copolymer Additive, Poly(l-lysine)-graft-poly(ethylene glycol). Tribol. Lett..

[50] Dedinaite A., Pettersson T., Mohanty B., Claesson P.M. (2010). Lubrication by organized soft matter. Soft Matter.

[51] Krivorotova T., Makuška R., Naderi A., Claesson P., Dėdinaitė A. (2010). Synthesis and interfacial properties of novel cationic polyelectrolytes with brush-on-brush structure of poly(ethylene oxide) side chains. Eur. Polym. J..

[52] Moglianetti M., Campbell R.A., Nylander T., Varga I., Mohanty B., Claesson P.M., Makuška R., Titmuss S. (2009). Interaction of sodium dodecyl sulfate and high charge density comb polymers at the silica/water interface. Soft Matter.

[53] Xu X., Billing M., Ruths M., Klok H., Yu J. (2018). Structure and Functionality of Polyelectrolyte Brushes: A Surface Force Perspective. Chem. Asian J..

[54] Mocny P., Klok H.-A. (2016). Tribology of surface-grafted polymer brushes. Mol. Syst. Des. Eng..

[55] Ma L., Gaisinskaya-Kipnis A., Kampf N., Klein J. (2015). Origins of hydration lubrication. Nat. Commun..

[56] Estrella R.P., Whitelock J.M., Packer N.H., Karlsson N.G. (2010). The glycosylation of human synovial lubricin: Implications for its role in inflammation. Biochem. J..

[57] Swann D.A., Sotman S., Dixon M., Brooks C. (1977). The isolation and partial characterization of the major glycoprotein (LGP-I) from the articular lubricating fraction from bovine synovial fluid. Biochem. J..

[58] Jay G.D., Waller K.A. (2014). The biology of Lubricin: Near frictionless joint motion. Matrix Biol..

[59] Pettersson T., Dėdinaitė A. (2008). Normal and friction forces between mucin and mucin–chitosan layers in absence and presence of SDS. J. Colloid Interface Sci..

[60] Efremova N.V., Huang Y., Peppas N.A., Leckband D.E. (2002). Direct Measurement of Interactions between Tethered Poly(ethylene glycol) Chains and Adsorbed Mucin Layers. Langmuir.

[61] Harvey N.M., Yakubov G.E., Stokes J.R., Klein J. (2011). Normal and Shear Forces between Surfaces Bearing Porcine Gastric Mucin, a High-Molecular-Weight Glycoprotein. Biomacromolecules.

[62] Zappone B., Patil N.J., Madsen J.B., Pakkanen K.I., Lee S. (2015). Molecular Structure and Equilibrium Forces of Bovine Submaxillary Mucin Adsorbed at a Solid–Liquid Interface. Langmuir.

[63] An J., Dėdinaitė A., Nilsson A., Holgersson J., Claesson P.M. (2014). Comparison of a Brush-with-Anchor and a Train-of-Brushes Mucin on Poly(methyl methacrylate) Surfaces: Adsorption, Surface Forces, and Friction. Biomacromolecules.

[64] An J., Jin C., Dėdinaitė A., Holgersson J., Karlsson N.G., Claesson P.M. (2017). Influence of Glycosylation on Interfacial Properties of Recombinant Mucins: Adsorption, Surface Forces, and Friction. Langmuir.

[65] Seog J., Dean D., Plaas A.H.K., Wong-Palms S., Grodzinsky A.J., Ortiz C. (2002). Direct Measurement of Glycosaminoglycan Intermolecular Interactions via High-Resolution Force Spectroscopy. Macromolecules.

[66] Hardingham T.E., Fosang A.J. (1992). Proteoglycans: Many forms and many functions. FASEB J..

[67] Han L., Dean D., Ortiz C., Grodzinsky A.J. (2007). Lateral Nanomechanics of Cartilage Aggrecan Macromolecules. Biophys. J..

[68] Horkay F., Basser P.J., Hecht A.-M., Geissler E. (2008). Gel-like behavior in aggrecan assemblies. J. Chem. Phys..

[69] Goldberg R., Schroeder A., Silbert G., Turjeman K., Barenholz Y., Klein J. (2011). Boundary Lubricants with Exceptionally Low Friction Coefficients Based on 2D Close-Packed Phosphatidylcholine Liposomes. Adv. Mater..

[70] Goldberg R., Schroeder A., Barenholz Y., Klein J. (2011). Interactions between Adsorbed Hydrogenated Soy Phosphatidylcholine (HSPC) Vesicles at Physiologically High Pressures and Salt Concentrations. Biophys. J..

[71] Goldberg R., Klein J. (2012). Liposomes as lubricants: Beyond drug delivery. Chem. Phys. Lipids.

[72] Sivan S., Schroeder A., Verberne G., Merkher Y., Diminsky D., Priev A., Maroudas A., Halperin G., Nitzan D., Etsion I. (2009). Liposomes Act as Effective Biolubricants for Friction Reduction in Human Synovial Joints. Langmuir.

[73] Dėdinaitė A., Wieland D.C.F., Bełdowski P., Claesson P.M. (2019). Biolubrication synergy: Hyaluronan–Phospholipid interactions at interfaces. Adv. Colloid Interface Sci..

[74] Cao Y., Kampf N., Klein J. (2019). Boundary Lubrication, Hemifusion, and Self-Healing of Binary Saturated and Monounsaturated Phosphatidylcholine Mixtures. Langmuir.

[75] Lin W., Kluzek M., Iuster N., Shimoni E., Kampf N., Goldberg R., Klein J. (2020). Cartilage-inspired, lipid-based boundary-lubricated hydrogels. Science.

[76] Tadmor R., Chen N., Israelachvili J. (2003). Normal and Shear Forces between Mica and Model Membrane Surfaces with Adsorbed Hyaluronan. Macromolecules.

[77] Li S., Macakova L., Bełdowski P., Claesson P.M., Dėdinaitė A. (2022). Phospholipids and Hyaluronan: From Molecular Interactions to Nano- and Macroscale Friction. Colloids Interfaces.

[78] Lapcik L., Lapcik L., De Smedt S., Demeester J., Chabreček P. (1998). Hyaluronan: Preparation, Structure, Properties, and Applications. Chem. Rev..

[79] Seror J., Merkher Y., Kampf N., Collinson L., Day A.J., Maroudas A., Klein J. (2011). Articular Cartilage Proteoglycans As Boundary Lubricants: Structure and Frictional Interaction of Surface-Attached Hyaluronan and Hyaluronan–Aggrecan Complexes. Biomacromolecules.

[80] Forsey R.W., Fisher J., Thompson J., Stone M.H., Bell C., Ingham E. (2006). The effect of hyaluronic acid and phospholipid based lubricants on friction within a human cartilage damage model. Biomaterials.

[81] Wang M., Liu C., Thormann E., Dėdinaitė A. (2013). Hyaluronan and Phospholipid Association in Biolubrication. Biomacromolecules.

[82] Raj A., Wang M., Zander T., Wieland D.F., Liu X., An J., Garamus V.M., Willumeit-Römer R., Fielden M., Claesson P.M. (2017). Lubrication synergy: Mixture of hyaluronan and dipalmitoylphosphatidylcholine (DPPC) vesicles. J. Colloid Interface Sci..

[83] Liu C., Wang M., An J., Thormann E., Dėdinaitė A. (2012). Hyaluronan and phospholipids in boundary lubrication. Soft Matter.

[84] Spahn G., Wittig R. (2003). Spannungs-und Bruchverhalten des gesunden Gelenkknorpels unter axialer Belastung. Eine biomechanische Untersuchung. Zent. Für Chir..

[85] Bełdowski P., Weber P., Dėdinaitė A., Claesson P.M., Gadomski A. (2018). Physical crosslinking of hyaluronic acid in the presence of phospholipids in an aqueous nano-environment. Soft Matter.

[86] Raj A., Wang M., Liu C., Ali L., Karlsson N.G., Claesson P.M., Dėdinaitė A. (2017). Molecular synergy in biolubrication: The role of cartilage oligomeric matrix protein (COMP) in surface-structuring of lubricin. J. Colloid Interface Sci..

[87] Dėdinaitė A., Claesson P.M. (2017). Synergies in lubrication. Phys. Chem. Chem. Phys..

[88] Yu J., Banquy X., Greene G., Lowrey D.D., Israelachvili J.N. (2011). The Boundary Lubrication of Chemically Grafted and Cross-Linked Hyaluronic Acid in Phosphate Buffered Saline and Lipid Solutions Measured by the Surface Forces Apparatus. Langmuir.

[89] Das S., Banquy X., Zappone B., Greene G.W., Jay G., Israelachvili J.N. (2013). Synergistic Interactions between Grafted Hyaluronic Acid and Lubricin Provide Enhanced Wear Protection and Lubrication. Biomacromolecules.

[90] Majd S.E., Kuijer R., Köwitsch A., Groth T., Schmidt T.A., Sharma P.K. (2014). Both Hyaluronan and Collagen Type II Keep Proteoglycan 4 (Lubricin) at the Cartilage Surface in a Condition That Provides Low Friction during Boundary Lubrication. Langmuir.

[91] Kelly M.T., Kent E.W., Zhao B. (2022). Stepwise Conformational Transitions of Stimuli-Responsive Linear Ternary Heterografted Bottlebrush Polymers in Aqueous Solution. Macromolecules.

[92] Li Z., Tang M., Liang S., Zhang M., Biesold G.M., He Y., Hao S.-M., Choi W., Liu Y., Peng J. (2021). Bottlebrush polymers: From controlled synthesis, self-assembly, properties to applications. Prog. Polym. Sci..

[93] Yin L., Liu L., Zhang N. (2021). Brush-like polymers: Design, synthesis and applications. Chem. Commun..

[94] Ribeiro J.P.M., Mendonça P.V., Coelho J.F.J., Matyjaszewski K., Serra A.C. (2020). Glycopolymer Brushes by Reversible Deactivation Radical Polymerization: Preparation, Applications, and Future Challenges. Polymers.

[95] Clarke B.R., Tew G.N. (2022). Synthesis and characterization of poly(ethylene glycol) bottlebrush networks via ring-opening metathesis polymerization. J. Appl. Polym. Sci..

[96] Wu Y., Tang Q., Zhang M., Li Z., Zhu W., Liu Z. (2018). Synthesis of bottlebrush polymers with v-shaped side chains. Polymer.

[97] Matyjaszewski K. (2018). Advanced Materials by Atom Transfer Radical Polymerization. Adv. Mater..

[98] Beers K.L., Gaynor S.G., Matyjaszewski K., Sheiko S.S., Möller M. (1998). The Synthesis of Densely Grafted Copolymers by Atom Transfer Radical Polymerization. Macromolecules.

[99] Lee H.-I., Boyce J.R., Nese A., Sheiko S.S., Matyjaszewski K. (2008). pH-induced conformational changes of loosely grafted molecular brushes containing poly(acrylic acid) side chains. Polymer.

[100] Chen Y., Zhou H., Sun Z., Li H., Huang H., Liu L., Chen Y. (2018). Shell of amphiphilic molecular bottlebrush matters as unimolecular micelle. Polymer.

[101] Cheng C., Khoshdel E., Wooley K.L. (2006). Facile One-Pot Synthesis of Brush Polymers through Tandem Catalysis Using Grubbs’ Catalyst for Both Ring-Opening Metathesis and Atom Transfer Radical Polymerizations. Nano Lett..

[102] Li Y., Themistou E., Zou J., Das B.P., Tsianou M., Cheng C. (2012). Facile Synthesis and Visualization of Janus Double-Brush Copolymers. ACS Macro Lett..

[103] Börner H.G., Beers A.K., Matyjaszewski K., Sheiko A.S.S., Möller M. (2001). Synthesis of Molecular Brushes with Block Copolymer Side Chains Using Atom Transfer Radical Polymerization. Macromolecules.

[104] Gao H., Matyjaszewski K. (2007). Synthesis of Molecular Brushes by “Grafting onto” Method: Combination of ATRP and Click Reactions. J. Am. Chem. Soc..

[105] Huang K., Canterbury D.P., Rzayev J. (2010). Organosoluble polypyrrole nanotubes from core–shell bottlebrush copolymers. Chem. Commun..

[106] Jin X.-H., Price M.B., Finnegan J.R., Boott C.E., Richter J.M., Rao A., Menke S.M., Friend R.H., Whittell G.R., Manners I. (2018). Long-range exciton transport in conjugated polymer nanofibers prepared by seeded growth. Science.

[107] Hua Z., Jones J.R., Thomas M., Arno M.C., Souslov A., Wilks T.R., O’Reilly R.K. (2019). Anisotropic polymer nanoparticles with controlled dimensions from the morphological transformation of isotropic seeds. Nat. Commun..

[108] Foster J.C., Varlas S., Couturaud B., Coe Z., O’Reilly R.K. (2019). Getting into Shape: Reflections on a New Generation of Cylindrical Nanostructures’ Self-Assembly Using Polymer Building Blocks. J. Am. Chem. Soc..

[109] Pearce A.K., Wilks T.R., Arno M.C., O’Reilly R.K. (2020). Synthesis and applications of anisotropic nanoparticles with precisely defined dimensions. Nat. Rev. Chem..

[110] Ma S., Zhang X., Yu B., Zhou F. (2019). Brushing up functional materials. NPG Asia Mater..

[111] Yang W., Zhou F. (2017). Polymer brushes for antibiofouling and lubrication. Biosurf. Biotribol..

[112] Lin W., Klein J. (2021). Recent Progress in Cartilage Lubrication. Adv. Mater..

[113] Chen M., Briscoe W.H., Armes S.P., Klein J. (2009). Lubrication at Physiological Pressures by Polyzwitterionic Brushes. Science.

[114] Wei Q., Cai M., Zhou F., Liu W. (2013). Dramatically Tuning Friction Using Responsive Polyelectrolyte Brushes. Macromolecules.

[115] Yu J., Jackson N.E., Xu X., Morgenstern Y., Kaufman Y., Ruths M., de Pablo J.J., Tirrell M. (2018). Multivalent counterions diminish the lubricity of polyelectrolyte brushes. Science.

[116] Banquy X., Burdyńska J., Lee D.W., Matyjaszewski K., Israelachvili J. (2014). Bioinspired Bottle-Brush Polymer Exhibits Low Friction and Amontons-like Behavior. J. Am. Chem. Soc..

[117] Ulcinas A., Valdre G., Snitka V., Miles M.J., Claesson P.M., Antognozzi M. (2011). Shear Response of Nanoconfined Water on Muscovite Mica: Role of Cations. Langmuir.

[118] Feuz L., Leermakers F.A.M., Textor M., Borisov O. (2008). Adsorption of Molecular Brushes with Polyelectrolyte Backbones onto Oppositely Charged Surfaces: A Self-Consistent Field Theory. Langmuir.

[119] Zappone B., Ruths M., Greene G., Jay G., Israelachvili J.N. (2007). Adsorption, Lubrication, and Wear of Lubricin on Model Surfaces: Polymer Brush-Like Behavior of a Glycoprotein. Biophys. J..

[120] Kang T., Banquy X., Heo J., Lim C., Lynd N.A., Lundberg P., Oh D.X., Lee H.-K., Hong Y.-K., Hwang D.S. (2016). Mussel-Inspired Anchoring of Polymer Loops That Provide Superior Surface Lubrication and Antifouling Properties. ACS Nano.

[121] Sedó J., Saiz-Poseu J., Busqué F., Ruiz-Molina D. (2013). Catechol-Based Biomimetic Functional Materials. Adv. Mater..

[122] Levine Z.A., Rapp M.V., Wei W., Mullen R.G., Wu C., Zerze G.H., Mittal J., Waite J.H., Israelachvili J.N., Shea J.-E. (2016). Surface force measurements and simulations of mussel-derived peptide adhesives on wet organic surfaces. Proc. Natl. Acad. Sci. USA.

[123] Lee S., Müller M., Heeb R., Zürcher S., Tosatti S., Heinrich M., Amstad F., Pechmann S., Spencer N.D. (2006). Self-healing behavior of a polyelectrolyte-based lubricant additive for aqueous lubrication of oxide materials. Tribol. Lett..

[124] Dobryden I., Steponavičiūtė M., Klimkevičius V., Makuška R., Dėdinaitė A., Liu X., Corkery R.W., Claesson P.M. (2019). Bioinspired Adhesion Polymers: Wear Resistance of Adsorption Layers. Langmuir.

[125] Dobryden I., Steponavičiūtė M., Hedman D., Klimkevičius V., Makuška R., Dėdinaitė A., Liu X., Corkery R.W., Claesson P.M. (2021). Local Wear of Catechol-Containing Diblock Copolymer Layers: Wear Volume, Stick–Slip, and Nanomechanical Changes. J. Phys. Chem. C.

[126] Drummond C., Marinov G., Richetti P. (2008). Reinforcement of a Surfactant Boundary Lubricant Film by a Hydrophilic−Hydrophilic Diblock Copolymer. Langmuir.

